# Building the basis for proteomics in personalized medicine for targeted treatment

**DOI:** 10.1186/s40169-016-0096-3

**Published:** 2016-05-20

**Authors:** Ho Jeong Kwon, Thomas E. Fehniger, György Marko-Varga

**Affiliations:** Chemical Genomics Global Research Lab, Department of Biotechnology, Translational Research Center for Function Control, College of Life Science and Biotechnology, Yonsei University, Seoul, 120-749 Republic of Korea; Department of Internal Medicine, Yonsei University College of Medicine, Seoul, 120-752 Republic of Korea; Clinical Protein Science & Imaging, Biomedical Center, Department of Biomedical Engineering, Lund University, BMC D13, SE-221 84 Lund, Sweden; Department of Surgery, Tokyo Medical University, 6-7-1 Nishishinjiku Shinjuku-ku, Tokyo, 60-0023 Japan

## Editorial

Today, genomics research, supported by protein expression studies, pave the way for the progress within molecular medicine. These developments deliver equally in pharmaceutical and biotech-industry R&D, as well as academic research. Proteomics, provides functional information concerning the activity of key regulating protein target(s) in disease, that the precision medicine has been developed for. As a progressive consequence, the proteomics impact reflected in the value of the global proteomics market has been predictable to have reached $17.2, by 2017. (http://www.spectroscopynow.com/details/news/140df70f7ba/.

Discovering biomarkers that predicts optimal treatment for patients is the holy grail of modern healthcare, which relates to the maximized efficacy that personalized medicine can provide. Early data, generated from large scale studies in lung cancer (non-small cell lung cancer, “NSCLC”), was proven to be of mandatory importance for predictive treatments of patients in Japan, as well as China and Korea [1–3]. These pioneering clinical studies, somatic mutation sequencing, complemented by proteomics profiling, later became a standard to Gefitinib (Iressa) developed by AstraZeneca. The drug agency, MLWH in Japan played an important role in these developments, requesting additional detailed data that linked the treatment of lung cancer patients to optimal efficacy and safety.

The introduction of Erlotinib (Tarceva) by Roche, as a targeted treatment in Japan followed the same strategy in predicative patient responders, as Iressa first outlined in Japan. Nowadays an entire range of small molecule drugs are available for cancer patients with tyrosine kinase inhibition (TKI) properties. GSK introduced Lapatinib, as a dual TKI, where HER2 and EGF pathway interruption occurs. As a consequence to all these developments, the MHLW presented a new strategy, where companion diagnostics are requested for new drugs that are introduced into the Japanese market. This strategy being implemented in Japan is to be expected to be followed as a basic healthcare principle worldwide.

These new practices have been complemented by the 4P medicine concept, introduced by Leroy Hood, (Institute for Systems Biology in Seattle), where proteomic multiplexing assays are included. The most recent 4P medicine Obama Healthcare initiated a “Big data” study, where 1 million patients will be incorporated with a total budget of $225 Mill. President Obama’s Precision Medicine Program with Leroy Hood has paved the way for the ability to predict and provide the right medicine to the right patient.

As the drug portfolio is increasing with safer and better products, national research programs are initiated globally to drive new initiatives to promote these developments. The Global Research Laboratory (GRL) project program in Korea is an example of an initiative for new technology developments to discover new chemical compound entities in nature products. Detailed drug action mechanisms are developed for medically active molecules isolated from plants and natural products [4, 5]. Understanding the mechanisms of drug action is key, in order to optimally design and develop drug molecules that provide optimal treatments for patients. It has to be emphasized that every new drug, being ordinated for a given patient involves a safety risk. These experiences have been developed over the last decade, especially in Cancer diseases, where some percentage of late stage patients will die during the treatment. These mortalities are most often associated by a combination of drug side effects and a complex pathology status of the patient. Imaging technologies by mass spectrometry is a growing science field, where both biomarkers in a pathological setting and drug mechanisms are being defined. Detailed molecular mechanisms and the metabolism of drugs are outlined as a part of the documentation requested by the authorities. Nowadays, we are able to provide MS imaging data on drug localization from a patient with a resolution at <10 µm. This resolving power provides data from a drug and metabolite, with a quantitative image-map at an intracellular organelle level. Mass spectrometry imaging is standard in Big-Pharna, where drug characteristics data requested by the FDA are determined. Fehniger and Marko-Varga were the first to introduce drug localization data in lung cancer-, and COPD- clinical studies, after drug administration [6, 7].

HUPO, the global human proteomics community enters fully engaged in projects that aim to create a better understanding of human biology and its complexities and to provide products from this new knowledge that will benefit humanity. We are heading towards a future, where high-quality standardized biomarkers of health, disease, response to therapy into the approval processes of regulatory agencies, e.g., U.S. Food and Drug Administration; FDA, and obtaining approval from the relevant agencies for their use in a clinical or other testing settings. To achieve this goal, its clear that the employment of standard processes for biobanking human clinical samples and enforcement of measures to ensure subject integrity, for the downstream use of samples.

It is envisaged that the increasing number of super-active drugs, such as personalized medicine for targeted treatment will foster new technology paradigms in mass spectrometry measurement. Proteomics platforms will open up new horizons in our understanding of protein expression, localization, interaction, structure, and physiological consequences important for patient treatment. There is especially an urgent need for a better understanding of target protein functions in human health and disease. The challenge is to find ways to provide necessary infrastructure and resources in proteomics, especially at the local level, to all members of the proteomics community.

Our proteomics community is actively responding to many of the goals and unmet needs of today’s society that is also supported by HUPO. We believe that these actions will build the foundation for providing solutions for meeting future needs. Finally, personalized medicine represents a paradigm shift with drug development research and therapy that changes patient treatments on a daily basis.
